# Cicadidae Periostracum, the Cast-Off Skin of Cicada, Protects Dopaminergic Neurons in a Model of Parkinson's Disease

**DOI:** 10.1155/2019/5797512

**Published:** 2019-10-24

**Authors:** Hye-Sun Lim, Joong-Sun Kim, Byeong Cheol Moon, Goya Choi, Seung Mok Ryu, Jun Lee, Mary Jasmin Ang, Mijin Jeon, Changjong Moon, Gunhyuk Park

**Affiliations:** ^1^Herbal Medicine Resources Research Center, Korea Institute of Oriental Medicine, 111 Geonjae-ro, Naju-si, Jeollanam-do 58245, Republic of Korea; ^2^College of Veterinary Medicine and BK21 Plus Project Team, Chonnam National University, Gwangju 61186, Republic of Korea

## Abstract

Parkinson's disease (PD) is characterized by dopaminergic neuronal loss in the substantia nigra pars compacta (SNPC) and the striatum. Nuclear receptor-related 1 protein (Nurr1) is a nuclear hormone receptor implicated in limiting mitochondrial dysfunction, apoptosis, and inflammation in the central nervous system and protecting dopaminergic neurons and a promising therapeutic target for PD. Cicadidae Periostracum (CP), the cast-off skin of *Cryptotympana pustulata* Fabricius, has been used in traditional medicine for its many clinical pharmacological effects, including the treatment of psychological symptoms in PD. However, scientific evidence for the use of CP in neurodegenerative diseases, including PD, is lacking. Here, we investigated the protective effects of CP on 1-methyl-4-phenyl-1,2,3,6-tetrahydropyridine- (MPTP-) induced PD in mice and explored the underlying mechanisms of action, focusing on Nurr1. CP increased the expression levels of Nurr1, tyrosine hydroxylase, DOPA decarboxylase, dopamine transporter, and vesicular monoamine transporter 2 via extracellular signal-regulated kinase phosphorylation in differentiated PC12 cells and the mouse SNPC. In MPTP-induced PD, CP promoted recovery from movement impairments. CP prevented dopamine depletion and protected against dopaminergic neuronal degradation via mitochondria-mediated apoptotic proteins such as B-cell lymphoma 2 (Bcl-2), Bcl-2-associated X, cytochrome c, and cleaved caspase-9 and caspase-3 by inhibiting MPTP-induced neuroinflammatory cytokines, inducible nitric oxide synthase, cyclooxygenase 2, and glial/microglial activation. Moreover, CP inhibited lipopolysaccharide-induced neuroinflammatory cytokines and response levels and glial/microglial activation in BV2 microglia and the mouse brain. Our findings suggest that CP might contribute to neuroprotective signaling by regulating neurotrophic factors primarily via Nurr1 signaling, neuroinflammation, and mitochondria-mediated apoptosis.

## 1. Introduction

Parkinson's disease (PD) is a progressive neurodegenerative disease characterized by bradykinesia, resting tremor, postural instability, and rigidity [1]. The disease affects 1–2% of the global population over the age of 65. In the brain of patients with PD, loss of dopamine-producing neurons in the substantia nigra pars compacta (SNPC) and the striatum (ST) may occur even prior to the onset of the symptoms of neurodegeneration [1, 2]. Available treatments work by relieving the symptoms of PD by increasing dopaminergic signaling through one of the three mechanisms: (1) increasing the dopamine levels by increasing the levels of its biosynthetic precursor (L-3,4-dihydroxyphenylalanine (L-DOPA)), (2) blocking the breakdown of dopamine by inhibiting its metabolic enzymes (monoamine oxidase, catechol-O-methyltransferase), and (3) mimicking the activity of dopamine by directly agonizing dopamine receptors [1, 3]. However, there is still an unmet clinical need to develop mechanism-based and/or disease-modifying medications to treat both the symptoms and progression of PD.

Nuclear receptor-related 1 protein (Nurr1) is a transcription factor that regulates the expression of genes that are critical for the development, maintenance, and survival of dopaminergic neurons [4, 5]. In particular, Nurr1 plays a fundamental role in maintaining dopamine homeostasis by regulating the transcription of genes governing dopamine synthesis, packaging, and reuptake [4]. Nurr1 also regulates the survival of dopaminergic neurons by stimulating the transcription of genes coding for neurotrophic factors, anti-inflammatory responses, and oxidative stress and mitochondrial dysfunction management, as well as repressing the transcription and expression of proinflammatory genes [4, 6, 7]. A lack of Nurr1 in embryonic ventral midbrain cells hinders their migration into striatal areas [8]. In microglia and astrocytes, Nurr1 represses proinflammatory responses and protects dopaminergic neurons from inflammation-induced neuronal toxicity or death in the midbrain [5, 9]. In patients with PD, the expression of Nurr1 is reduced compared to age-matched controls, and a few, yet rare, Nurr1 polymorphisms appear to be associated with the disease [10, 11]. Stimulation of Nurr1 activity may combat both the reduced dopamine levels and the increased oxidative stress and inflammation associated with PD [12–14]. Together, these findings strongly suggest that disrupted function/expression of Nurr1 is related to neurodegeneration of dopaminergic neurons and alleviates inflammation and mitochondrial dysfunctions; thereby, it may improve the pathogenesis of PD.

Cicadidae Periostracum (CP), the cast-off skin of *Cryptotympana pustulata* Fabricius (also known as cicada or Sun-Tae), was originally described in the Chung-bu category of *Dongui Bogam*, an ancient Korean medical book [15, 16]. In Korean traditional medicine, CP has been used to treat epilepsy, shock, smallpox, sedation, edema, and night terror symptoms. In traditional Chinese medicine, CP is known as chantui and has long been used to treat soreness of the throat, hoarseness, itching, spasms, and other symptoms [17]. Since then, it has been used in traditional medicine for its many pharmacological effects. In 2003, a World Health Organization (WHO) international expert meeting to review and analyze the clinical reports on severe acute respiratory syndrome (SARS) treatment noted that the Chinese were using a combination of cicada and silkworm droppings to treat SARS-associated fever [18, 19]. Recently, several studies have confirmed the pharmacological effects of CP, including its anti-skin aging, anti-kidney injury, anticonvulsive, sedative, antiallergy, and antianaphylactic shock actions [20–23]. However, scientific evidence for the use of CP in neurodegenerative diseases, including PD, is lacking. Therefore, in this study, we investigated the protective effects of CP on 1-methyl-4-phenyl-1,2,3,6-tetrahydropyridine- (MPTP-) induced neurotoxicity in mice and explored the underlying mechanisms of action, focusing on Nurr1.

## 2. Materials and Methods

### 2.1. Preparation of the CP Extract

CP was purchased from Kwong-Mung-dang Company (Ulsan, Korea) and authenticated by Dr. Goya Choi (Herbal Medicine Resources Research Center, Korea Institute of Oriental Medicine, Naju, Korea), and a voucher specimen (3-18-0038) was deposited at the Herbal Medicine Resources Research Center, Korea Institute of Oriental Medicine. Briefly, CP was extracted in distilled water for 3 h under reflux (100 ± 2°C). Then, the extract was filtered, evaporated on a rotary vacuum evaporator, and lyophilized (yield, 6.30%). The powder was kept at 4°C until use.

### 2.2. Animals

Male C57BL/6 mice (8 weeks, 23–24 g) were purchased from Doo Yeol Biotech (Seoul, Korea) and maintained under temperature- and light-controlled conditions (20–23°C, 12 h light/12 h dark cycle) with food and water provided ad libitum. All animals were acclimatized for 7 days prior to drug administration. The experimental protocol was approved by the institutional animal care committee of KIOM (KIOM-18-056) and performed according to the guidelines of the Animal Care and Use Committee at KIOM.

### 2.3. Drug Administration

**Figure 1:**
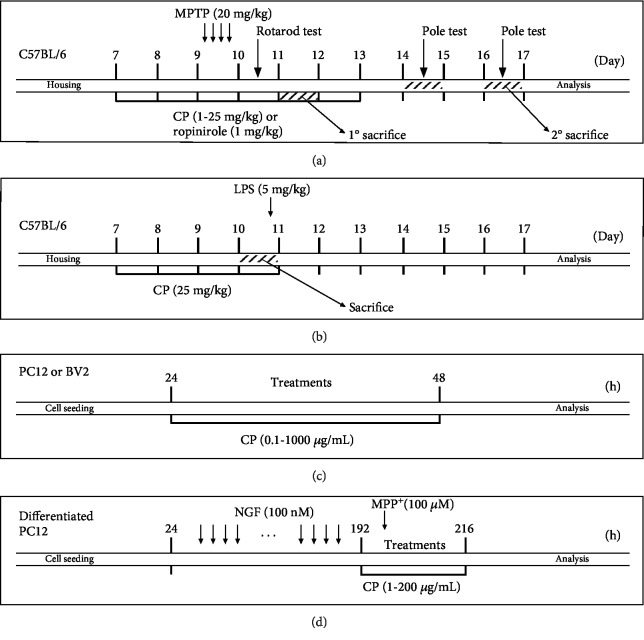
Summary of the experimental design.

Mice were assigned to 1 of 11 groups: (1) control (*n* = 11), (2) MPTP (*n* = 11), (3) MPTP+CP 1 mg/kg/day (*n* = 11), (4) MPTP+CP 10 mg/kg/day (*n* = 11), (5) MPTP+CP 25 mg/kg/day (*n* = 11), (6) MPTP+ropinirole 1 mg/kg/day (*n* = 11), (7) CP 5 mg/kg/day (*n* = 5), (8) CP 25 mg/kg/day (*n* = 5), (9) control (*n* = 7), (10) lipopolysaccharide (LPS, *n* = 7), and (11) LPS+CP 25 mg/kg/day (*n* = 7). CP, dissolved in normal saline, was administered for 5 days consecutively. The control group received an equal volume of normal saline for the same duration. MPTP (Sigma-Aldrich, St. Louis, MO, USA) or LPS (Sigma-Aldrich) were administered acutely as described previously [24–29]. On day 3 of CP treatment, MPTP (20 mg/kg, dissolved in saline) was injected intraperitoneally four times at 2 h intervals. Vehicles of equal volume (0.25 mL) were given to the control group (Figure 1(a)). In the LPS group, 3 h after the last administration of CP, LPS was dissolved in saline and injected intraperitoneally at a dose of 5 mg/kg (Figure 1(b)). Since MPTP is a very dangerous chemical, we conducted the experiment in compliance with previously described procedures [30]: (1) we used laboratory clothing and boots made of nonabsorbable material, (2) we wore a mask with a HEPA filter, (3) 1% bleach was used to clean all equipment used in the experiments, and (4) all waste was disposed in a biohazard safety bin.

### 2.4. Rotarod Test and Pole Test

The rotarod test and pole test were assessed according to previously published methods [25, 29, 31]. The rotarod test is a useful method for measuring hypokinesia in a mouse model of PD [32]. Mice were evaluated on the rotarod 1 day after the last MPTP injection to assess sensorimotor coordination. The rotarod unit (Ugo Basile, Comerio, Varese, Italy) consisted of a rotating spindle (diameter 3 cm) and five individual compartments, which allowed five mice to be tested simultaneously. After two successive days of twice-daily training (4 rpm rotation speed on the first day and 20 rpm on the second day), the test rotation speed was increased to 25 rpm on the third day in a test session. The time each mouse remained on the rotating bar was recorded over three trials per mouse, at 5 min intervals and a maximum trial length of 300 s per trial. Data are presented as mean time on the rotating bar over the three test trials.

The pole test is a useful method to measure bradykinesia in a mouse model of PD [32]. We performed the pole test on days 5 and 7 after the last MPTP injection. The mice were held on the top of the pole (diameter 8 mm, height 55 cm, with a rough surface). The time that mice needed to turn down completely was recorded as the time to turn (T-turn). The time needed for the mice to climb down and place four feet on the floor was recorded as the time for locomotion activity (T-LA). Each trial had a cut-off limit of 50 s.

### 2.5. Brain Tissue Preparation

On days one and seven after MPTP treatment or at 3 h after LPS treatment, mice were anesthetized immediately and perfused transcardially with 0.05 M phosphate-buffered saline (PBS, Sigma-Aldrich), followed by cold 4% paraformaldehyde (PFA, Sigma-Aldrich) in 0.1 M phosphate buffer. Brains were removed and postfixed in 0.1 M phosphate buffer (Sigma-Aldrich) containing 4% PFA overnight at 4°C and then immersed in a solution containing 30% sucrose in 0.05 M PBS for cryoprotection. Serial 15 or 30 *μ*m thick coronal sections were cut on a freezing microtome (Leica Instruments GmbH, Nussloch, Germany) and stored in a cryoprotectant (25% ethylene glycol, Sigma-Aldrich), 25% glycerol (Sigma-Aldrich), and 0.05 M phosphate buffer at 4°C until use for immunohistochemistry (IHC) study. For western blotting mRNA analysis, and kit-based analyses, in brief, the SNPC and ST were rapidly dissected, homogenized, and centrifuged using standard laboratory techniques. The final supernatant was stored at 70°C until use [33].

### 2.6. Immunohistochemistry (IHC) and Immunofluorescence Analysis

For IHC analysis, the assessment of dopaminergic neurons in the SNPC was made by analyzing coronal sections at approximately 3.28 mm behind the bregma [34]. Brain sections were rinsed briefly in PBS and treated with 1% hydrogen peroxide (Sigma-Aldrich) for 15 min. The sections were incubated with rabbit anti-tyrosine hydroxylase (TH) (1 : 1000) overnight at 4°C in the presence of 0.3% Triton X-100 (Vector Laboratories, Burlingame, CA, USA) and normal goat serum (Vector Laboratories) or normal horse serum (Vector Laboratories). After rinsing in PBS, the sections were incubated with biotinylated anti-rabbit IgG (Vector Laboratories) (1 : 200) for 90 min, rinsed, and incubated with ABC (Vector Laboratories) (1 : 100) for 1 h at room temperature. Peroxidase activity was visualized by incubating sections with DAB (Sigma-Aldrich) in 0.05 M Tris-buffered saline (Sigma-Aldrich). After several rinses with PBS, sections were mounted on gelatin-coated slides, dehydrated, and cover-slipped using histomount medium. For immunofluorescence analysis, brain sections were rinsed briefly in PBS and treated with 0.5% BSA (Sigma-Aldrich) for 30 min. The sections were incubated with mouse anti-TH (1 : 100) or rabbit anti-glial fibrillary acidic protein (GFAP), ionized calcium-binding adaptor molecule 1 (Iba-1), Nurr1, or cyclooxygenase (Cox)-2 (1 : 200) overnight at 4°C in the presence of 0.3% Triton X-100 and normal serum. Next, they were incubated for 1 h with Alexa Fluor-conjugated secondary antibodies (1 : 500). The sections were finally washed in PBS and mounted using Vectashield mounting medium containing DAPI (Vector Laboratories). Images were photographed at 40x and 100x magnification using an optical light microscope (Olympus Microscope System BX53; Olympus, Tokyo, Japan) equipped with a 20x objective lens. Further, to analyze the intensity of striatal TH-positive nerve fibers, brain sections were sampled at approximately 0.62 mm ahead of the bregma. Primary antibodies are listed in Supplementary [Supplementary-material supplementary-material-1].

### 2.7. RNA Extraction and Real-Time Reverse Transcription Polymerase Chain Reaction

Homogenization of SNPC tissue was conducted using TRIzol reagent (Invitrogen, Carlsbad, CA, USA). After homogenization, 0.2 mL of chloroform was added to each sample. The tubes were shaken vigorously by hand for 15 s and then incubated at room temperature for 3 min. Next, the mixture was centrifuged at 14,000 rpm for 15 min at 4°C, after which the resulting upper aqueous phase (400 *μ*L) was transferred to a fresh tube into which 0.5 mL of 2-propanol was also added. After incubation for 10 min at 4°C, the mixture was centrifuged again at 14,000 rpm for 10 min at 4°C. After separation, the supernatant was removed, washed with 1 mL of 75% ethanol, and centrifuged again at 10,000 rpm for 5 min at 4°C. The resulting RNA pellet was then dried, and the purified RNA was dissolved in diethyl pyrocarbonate- (DEPC-) distilled water. Equal amounts of RNA (200 ng) were reverse transcribed (RT) into cDNA using an iScript cDNA synthesis kit (Bio-Rad, Hercules, CA, United States) according to the manufacturer's protocol. Real-time RT polymerase chain reaction (PCR) analysis was performed for selected genes using the CFX96 real-time PCR system (Bio-Rad) and the SYBR green fluorescence quantification system (Bio-Rad) to quantify the amplicons. The PCR conditions were 50 cycles of 95°C (30 s) and 58°C (30 s) and a standard denaturation curve. The primer sequences are listed in the 5′ to 3′ orientation in Supplementary [Supplementary-material supplementary-material-1]. The PCR conditions for each target were optimized according to the primer concentration, the absence of primer dimer formation, and the efficiency of amplification of both the target genes and the housekeeping control gene. PCR reaction mixture comprised 1 *μ*L of cDNA and 9.5 *μ*L of PCR master mix, which contained 2× SYBR Green, 10 pmol each of the forward and reverse primers, and 4.5 *μ*L of DEPC-distilled water in a final volume of 15 *μ*L. To normalize the cDNA content of the samples, we used the comparative threshold (CT) cycle method, which includes normalization of the number of target gene copies vs. the endogenous reference gene glyceraldehyde 3-phosphate dehydrogenase. The CT is defined as the fractional cycle number at which the fluorescence generated by cleavage of the probe passes a fixed threshold baseline when amplification of the PCR products is first detected.

### 2.8. Differentiated PC12 Cell and Microglial BV2 Cell Culture

The pheochromocytoma PC12 cell line, derived from the rat adrenal medulla, was obtained from the Korean Cell Line Bank (#21721). PC12 cells were maintained in Roswell Park Memorial Institute medium (Gibco, MD, USA) supplemented with 10% heat-inactivated fetal bovine serum (Gibco) and 1% penicillin/streptomycin (Gibco) in an atmosphere of 95% air and 5% CO_2_ at 37°C. The PC12 cells were seeded on 96-well plates or 6-well plates at a density of 1 or 2 × 10^5^ cells/mL and treated with CP (0–1000 *μ*g/mL) for 24 h (Figure 1(c)). For differentiated PC12, culturing media were renewed every 2 to 3 days. Cultured PC12 cells were treated with nerve growth factor (Sigma-Aldrich) for 7 days, with fresh medium and reagents supplied every 24 h. The differentiated PC12 cells were treated with CP (1–200 *μ*g/mL) for 1 h. Then, they were stimulated with MPP^+^ (100 *μ*M) for an additional 23 h (Figure 1(d)).

The mouse microglial BV2 cell line was kindly donated by Prof. Jung at Seoul National University Hospital in Korea. The cells were kept in Dulbecco's modified Eagle's medium (Gibco) supplemented with 10% heat-inactivated fetal bovine serum and 1% penicillin/streptomycin in an atmosphere of 95% air and 5% CO_2_ at 37°C. The BV2 cells were seeded on 24-well plates at a density of 1.5 × 10^5^ cells/mL and treated with CP (0–1000 *μ*g/mL) for 24 h (Figure 1(c)).

### 2.9. Measurement of Living Cells

Living cells were evaluated using the Cell Counting Kit (CCK-8; Dojindo, Kumamoto, Japan) according to the manufacturer's protocol. In brief, cells were seeded on 96-well plates and treated with CP. The CCK-8 reagent was added to each well, and the mixture was incubated for 4 h. The absorbance was read at 450 nm using the SpectraMax i3 Multi-Mode Detection Platform (Molecular Devices, Sunnyvale, CA, USA). Cell viability was calculated using the following equation: %of living cells left after CP treatment = (mean absorbance in CP − treated cells/mean absorbance in untreated controls) × 100.

### 2.10. Western Blotting Analysis and Measurement of Mitochondrial Membrane Potential

Western blotting was performed according to previously published methods [28, 35, 36]. Moreover, Δ*ψ*m was monitored with the lipophilic cationic probe tetraethylbenzimidazolylcarbocyanine iodide (JC-1) reagent, supplied with the Δ*ψ*m detection kit, according to the manufacturer's instructions. Differentiated PC12 cells were seeded on coverslips in 96-well plates and treated simultaneously with CP at concentrations of 1, 10, or 50 *μ*g/mL and 100 *μ*M MPP^+^ for 24 h. Then, the cells were rinsed with PBS and incubated with the diluted lipophilic cationic probe 5,5′,6,6′-tetrachloro-1,1′,3,3′-tetraethylbenzimidazol-carbocyanine iodide (JC-1) at 37°C for 30 min, after which the cells were washed and transferred to 96-well plates. The ratio of red (585/590 nm) and green (510/527 nm) fluorescence was determined using a fluorescence plate reader.

### 2.11. Small Interfering RNA Transfection

Differentiated PC12 cells were used at a confluence of 80–85% in 100 mm dishes. Cells were transfected with stealth small interfering RNA (siRNA) using Lipofectamine 2000 (Invitrogen). Lipofectamine 2000 (10 *μ*L) was mixed with 40 *μ*M siRNA solution (an equimolar mix of both Nurr1 siRNA and scrambled siRNA) and 2.5 mL of Opti-MEM (Gibco). After 30 min at room temperature, 300 *μ*L of the mix was added to 300 *μ*L of serum-free RPMI in each dish and incubated for 24 h.

### 2.12. Dopamine and Cytokine Array Levels

The dopamine contents in the ST of the mouse brain were assessed using a commercially available fluorometric assay kit, following the protocol supplied by the manufacturer (Rocky Mountain Diagnostics). Moreover, cytokine proteins were determined using a cytokine membrane array kit following the manufacturer's instructions (R&D Systems, Minneapolis, MN, USA).

### 2.13. Statistical Analyses

All statistical parameters were calculated using the GraphPad Prism 7.0 software (GraphPad Software, San Diego, CA, USA). Values are expressed as means ± standard error of the mean (S.E.M.). Statistical comparisons between the different treatments were performed using one-way analysis of variance (ANOVA) with Tukey's multiple comparison posttest. A *p* value < 0.05 was considered to be statistically significant.

## 3. Results

### 3.1. Effect of CP on MPTP-Induced Movement Impairment

**Figure 2:**
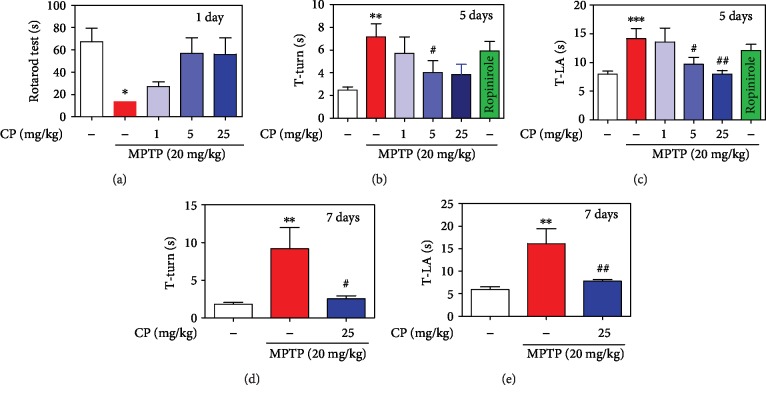
Effects of CP on MPTP-induced movement impairment in mice. CP was administered for 5 days. On day 3, at 2 h after CP administration, MPTP was injected four times. One day after MPTP injection, latency time on the rotating rod was recorded with a 300 s cut-off limit (a). At days 5 and 7 after MPTP injection, time to turn completely downward (b, d) and time to fall off the rod onto the floor (c, e) were recorded with a 60 s cut-off limit. Values shown represent means ± S.E.M. ^∗^*p* < 0.05, ^∗∗^*p* < 0.01, and ^∗∗∗^*p* < 0.001 compared with the control group and ^#^*p* < 0.05 and ^##^*p* < 0.01 compared with the MPTP-treated group.

To examine the effect of CP on MPTP-induced poor motor coordination and postural balance, a rotarod test was performed. We found that MPTP significantly decreased the retention time to 13.71 ± 2.06 s on day 1, compared with the control. However, retention times were significantly increased in the MPTP+1–25 mg/kg/day CP and ropinirole groups from 27.14 ± 4.31 to 56.86 ± 13.99 s and 55.86 ± 14.81 s, respectively, on day 1 (Figure 2(a)). In addition, to evaluate the effects of CP on MPTP-induced bradykinesia, a pole test was performed. T-turn and T-LA were significantly prolonged to 7.17 ± 1.10s and 14.13 ± 1.72 s, respectively, on day 5, compared with the control. However, T-turn was significantly shortened in the MPTP+1–25 mg/kg/day CP and ropinirole groups from 5.70 ± 1.41 to 3.83 ± 0.76 s and 5.91 ± 0.81 s, respectively, on day 5. However, T-LA was significantly shortened in the MPTP+1–25 mg/kg/day CP and ropinirole groups from 13.58 ± 2.34 to 7.97 ± 0.63 s and 12.09 ± 1.02 s, respectively, on day 5 (Figures 2(b) and 2(c)). T-turn and T-LA were significantly prolonged to 9.18 ± 2.23 s and 16.12 ± 2.66 s, respectively, on day 7, compared with the control. However, T-turn and T-LA were significantly shortened in the MPTP 25 mg/kg/day CP groups to 2.52 ± 0.40 s and 7.79 ± 0.40 s, respectively, on day 7 (Figures 2(d) and 2(e)).

### 3.2. Effects of CP on MPTP-Induced Dopaminergic Neuronal Loss and Dopamine Depletion

**Figure 3:**
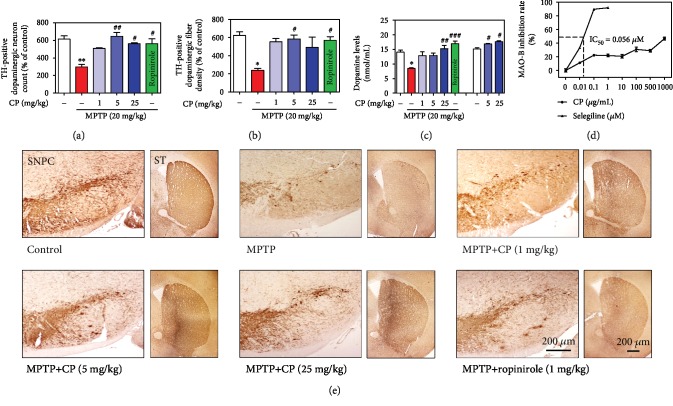
Effects of CP on MPTP-induced dopaminergic neuronal death. Dopaminergic neurons were visualized by TH-specific immunostaining. The number of TH-immunopositive neurons in the SNPC (a) was counted, and the optical density in the ST (b) was measured. Dopamine levels in the ST were measured using ELISA (c). MAO-B inhibition rate in a cell-free system was measured using ELISA (d). Representative photomicrographs of the SNPC and ST were taken (e). Values are presented as means ± S.E.M. ^∗^*p* < 0.05, ^∗∗^*p* < 0.01, and ^∗∗∗^*p* < 0.001 compared with the control group and ^#^*p* < 0.05, ^##^*p* < 0.01, and ^###^*p* < 0.001 compared with the MPTP-treated group (a, b, or c) or control group (c in the cell-free system).

To confirm the effects of CP on dopaminergic neuronal death, we performed TH-specific immunohistochemistry in the SNPC and ST of mouse brains. In MPTP-treated mice, the number of TH-positive cells in the SNPC and the optical intensity in the ST were decreased by 48.51 ± 3.85% and 38.20 ± 3.37%, respectively, compared with the control group. However, these values were significantly increased by 1–25 mg/kg CP or ropinirole treatment (82.64 ± 1.31% to 91.50 ± 1.31% and 91.61 ± 7.30% and 88.88 ± 4.75% to 79.01 ± 18.21% and 91.64 ± 6.30%, respectively, compared with the control group) (Figures 3(a), 3(b), and 3(e)). Moreover, to measure the effects of CP on dopamine levels, we determined striatal dopamine levels in the ST of mouse brains (Figure 3(c)). Treatment with MPTP significantly decreased striatal dopamine (by 8.47 ± 0.25 nmol/mL) compared with the control group, while treatment with 1–25 mg/kg CP or ropinirole reduced MPTP-induced striatal dopamine (by 12.84 ± 1.36 to 15.20 ± 1.18 nmol/mL and 16.94 ± 0.93 nmol/mL). Moreover, only the treatment with 5–25 mg/kg CP increased striatal dopamine (by 16.86 ± 0.21 to 17.65 ± 0.29 nmol/mL) compared with the control group (Figure 3(c)). We also investigated the effect of CP on MAO-B activity. Selegiline (positive control, 0–1 *μ*M) inhibited MAO-B activity in a dose-dependent manner; in contrast, CP had no effect on MAO-B activity (Figure 3(d)).

### 3.3. Effects of CP on the Induction of Nurr1 and Its Regulating Proteins and Dopamine Depletion

**Figure 4:**
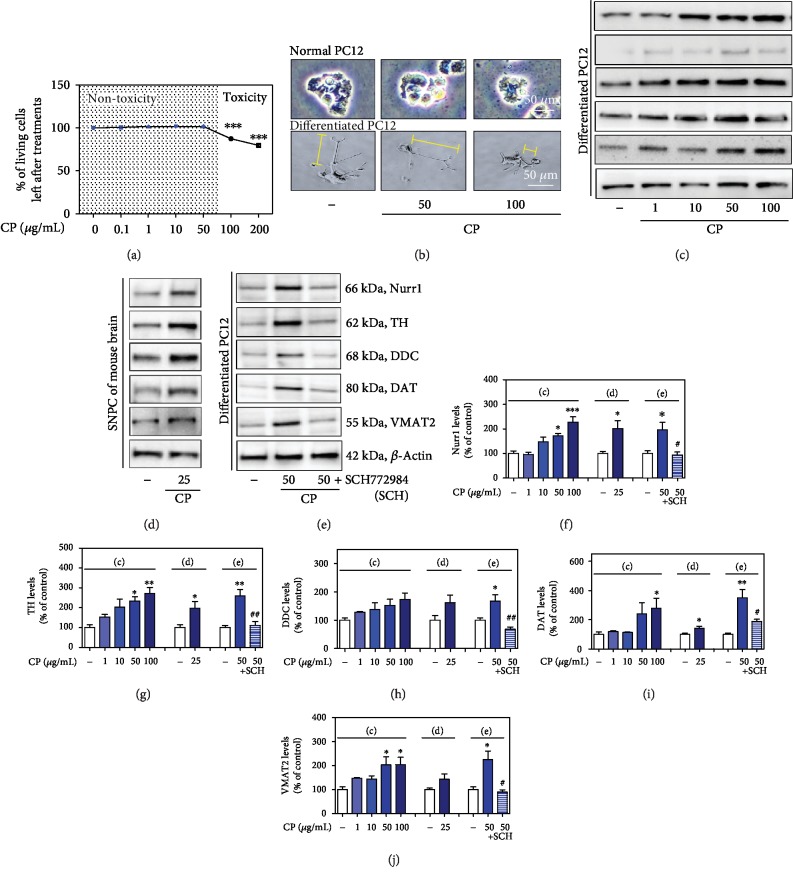
Effects of CP on cell viability and Nurr1 and its regulating neurotrophic factors. Effects of CP on PC12 and differentiated PC12 cell neuronal damage (a, b). The protein levels of Nurr1 and its regulating neurotrophic factors were measured by western blotting in differentiated PC12 cells (c) and the mouse SNPC (d). Differentiated PC12 cells were pretreated with CP and an ERK inhibitor (SCH) for 10 h, and the protein levels of Nurr1 and its regulating neurotrophic factors were measured by western blotting (e). *β*-Actin protein was used as an internal control. Bar graphs represent the relative expression of Nurr1 (f), TH (g), DDC (h), DAT (i), and VMAT2 (j) for (c)–(e). Values are presented as means ± S.E.M. ^∗^*p* < 0.05, ^∗∗^*p* < 0.01, and ^∗∗∗^*p* < 0.001 compared with the control group and ^#^*p* < 0.05 and ^##^*p* < 0.01 compared with the CP-treated group.

Treatment with CP at 100 *μ*g/mL, but not at 1–50 *μ*g/mL, increased neuronal shrinkage and damage and decreased dendritic length of PC12 and differentiated PC12 cells 24 h after treatment (Figures 4(a) and 4(b)). Thus, all further experiments were performed with CP at 1–50 *μ*g/mL. To investigate the effects of CP on Nurr1 and its regulating proteins, we determined the levels of Nurr1, TH, DDC, DAT, and VMAT2 in differentiated PC12 cells. The protein expression levels of Nurr1, TH, DDC, DAT, and VMAT2 were increased 20 h after CP treatment (Nurr1: 95.42 ± 8.31% to 226.96 ± 22.30%, TH: 152.26 ± 14.77% to 272.45 ± 29.81%, DDC: 128.03 ± 2.89% to 173.43 ± 22.66%, DAT: 118.17 ± 6.47% to 278.32 ± 29.55%, and VMAT2: 147.26 ± 3.32% to 203.69 ± 31.21% compared with the control group) (Figures 4(c) and 4(f)–4(j)). We confirmed the effects of CP on Nurr1 and its regulating proteins in the mouse SNPC (Nurr1: 201.02 ± 32.55%, TH: 197.50 ± 33.64%, DDC: 161.74 ± 27.33%, DAT: 140.97 ± 14.67%, and VMAT2: 142.77 ± 22.56% compared with the control group) (Figures 4(d) and 4(f)–4(j)).

To investigate whether ERK activation contributed to CP-induced increased Nurr1 expression, we used SCH772984, a selective inhibitor of ERK. SCH772984 inhibited a CP-induced increase in Nurr1, TH, DDC, DAT, and VMAT2 in differentiated PC12 cells (Nurr1: 196.24 ± 30.19% and 94.31 ± 11.10%, TH: 258.87 ± 33.33% and 111.47 ± 19.10%, DDC: 166.88 ± 23.84% and 68.21 ± 7.94%, DAT: 350.47 ± 56.53% and 187.24 ± 16.89%, and VMAT2: 224.09 ± 36.33% and 89.09 ± 10.16% compared with the control group) (Figures 4(e) and 4(f)–4(j)).

### 3.4. Effects of CP on MPTP-Induced Nurr1 Expression in Dopaminergic Neurons

**Figure 5:**
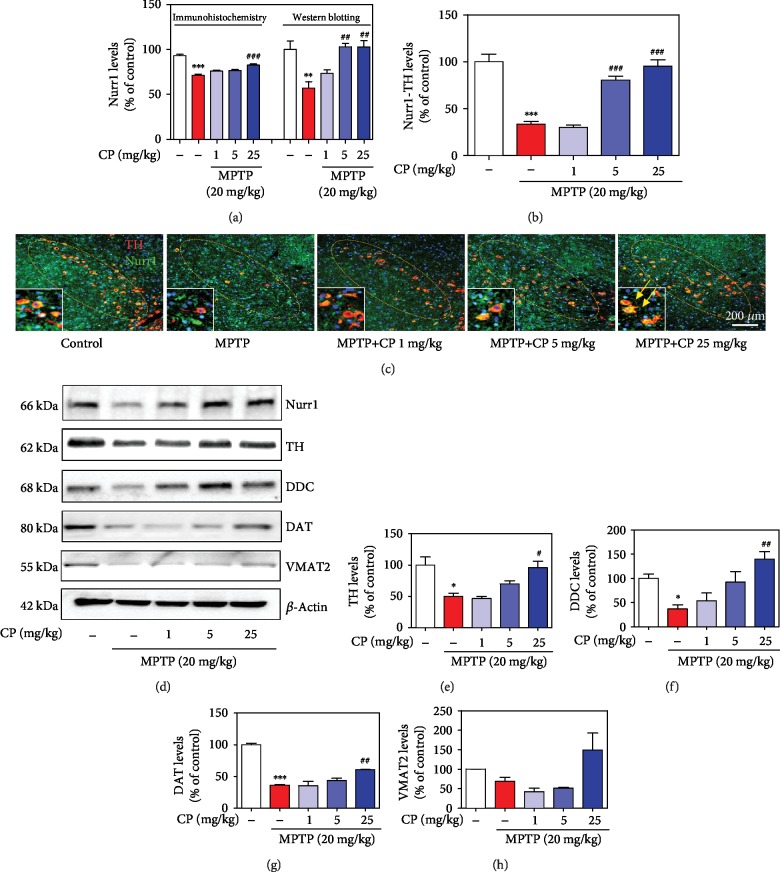
Effects of CP on MPTP-induced Nurr1 and neurotrophic proteins. Seven days after the last MPTP treatment, Nurr1 levels were measured by IHC in dopaminergic neurons (a, b) and western blotting in the SNPC (a). Representative photomicrographs of the SNPC were taken (c). The expressions of Nurr1, TH, DDC, DAT, and VMAT2 were detected by western blotting using specific antibodies in the SNPC (d). *β*-Actin protein was used as an internal control. Bar graphs represent the relative expression of Nurr1 (a), TH (e), DDC (f), DAT (g), and VMAT2 (h) for (d). Values shown represent means ± S.E.M. ^∗^*p* < 0.05 and ^∗∗∗^*p* < 0.001 compared with the control group and ^#^*p* < 0.05 and ^##^*p* < 0.01 compared with the MPTP-treated group.

To evaluate the effects of CP on Nurr1 expression, we performed Nurr1-specific immunofluorescence staining and western blotting. MPTP significantly decreased Nurr1 (by 71.49 ± 1.27%) in shrunken dopaminergic neurons (by 33.40 ± 3.17%) compared with the control, and treatment with 5–25 mg/kg CP reduced MPTP-induced Nurr1 (by 76.08 ± 1.15% to 82.67 ± 1.30%) and Nurr1 in dopaminergic neurons further (by 30.06 ± 2.40% to 95.06 ± 7.22%) (Figures 5(a)–5(c)). Moreover, we confirmed Nurr1 expression levels by western blotting, and MPTP significantly decreased Nurr1 (by 33.40 ± 3.13%) in the SNPC, compared with the control, and treatment with 5–25 mg/kg CP reduced MPTP-induced Nurr1 (by 30.06 ± 2.34% to 95.06 ± 7.07%) (Figures 5(a)–5(d)). Representative photomicrographs were taken of double-labeled immunofluorescence staining with anti-Nurr1 and anti-TH antibodies in the SNPC (Figure 5(c) and Supplementary Fig. [Supplementary-material supplementary-material-1]).

### 3.5. Effects of CP on MPTP-Induced Expression of Nurr1 Regulating Proteins

**Figure 6:**
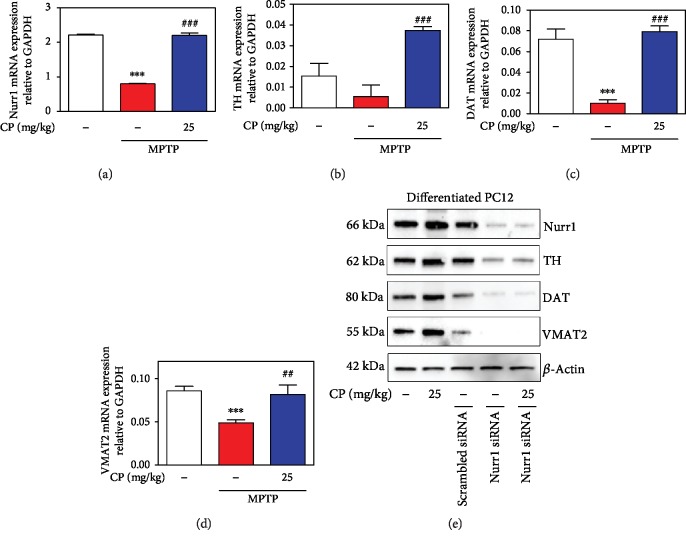
Effects of CP on MPTP-induced mRNA levels of Nurr1 and its regulating neurotrophic factors. Real-time RT-PCR was performed to see the effects of CP on mRNA expression of Nurr1 (a), TH (b), DAT (c), and VMAT2 (d). Then, effects of Nurr1 on upregulating neurotrophic factors (TH, DAT, and VMAT2) in Nurr1 siRNA-transfected differentiated PC12 cells (e). Values shown represent means ± S.E.M. ^∗∗∗^*p* < 0.001 compared with the control group and ^##^*p* < 0.01 and ^###^*p* < 0.001 compared with the MPTP-treated group.

To evaluate the effects of CP on MPTP-induced expression of Nurr1 and its regulating proteins, we assessed the levels of TH, DDC, DAT, and VMAT2 in the mouse SNPC by western blotting. MPTP significantly decreased TH (by 49.87 ± 5.21%), DDC (by 36.73 ± 8.28%), DAT (by 36.17 ± 0.97%), and VMAT2 (by 69.12 ± 10.67%) levels, compared with the control, while treatment with 1–25 mg/kg CP reduced MPTP-induced expression of TH (by 46.31 ± 3.94% to 95.71 ± 10.59%), DDC (by 53.45 ± 16.29% to 140.27 ± 15.04%), DAT (by 34.98 ± 7.3% to 60.85 ± 1.08%), and VMAT2 (by 42.14 ± 9.55% to 149.34 ± 43.42%), compared with the control group (Figures 5(d)–5(h)). In addition, we measured the mRNA expression levels of Nurr1 and its regulating proteins using real-time RT-PCR analysis. MPTP significantly decreased Nurr1 (by 0.82 ± 0.01), TH (by 0.01 ± 0.01), DAT (by 0.01 ± 0.003), and VMAT2 (by 0.05 ± 0.003) levels, while treatment with 25 mg/kg CP reduced MPTP-induced expression of Nurr1 (by 2.20 ± 0.08), TH (by 0.04 ± 0.001), DAT (by 0.08 ± 0.01), and VMAT2 (by 0.08 ± 0.01) (Figures 6(a)–6(d)). Moreover, to further verify whether CP-induced TH, DAT, and VMAT2 were mediated through Nurr1 activation, we transfected differentiated PC12 cells with siRNA targeting Nurr1. Results showed that TH, DAT, and VMAT2 levels were not affected by CP in Nurr1 siRNA-transfected cells (Figure 6(e)).

### 3.6. Effects of CP on MPTP-Induced Mitochondrial Dysfunction and Mitochondria-Mediated Apoptosis

**Figure 7:**
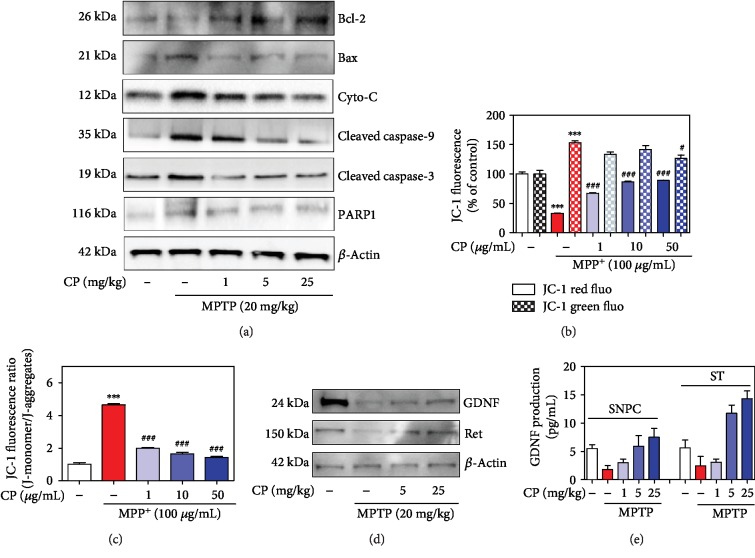
Effects of CP on MPTP-induced mitochondria-mediated apoptosis. Seven days after the last MPTP treatment, mitochondria-induced apoptotic factors (Bcl-2, Bax, Cyt-c, cleaved caspase-9, cleaved caspase-3, and PARP1) were measured by western blotting (a). Differentiated PC12 cells were treated with CP and exposed to MPP^+^ for 48 h. Moreover, red and green (b) and the ratio (c) of these fluoresces of mitochondrial membrane potential were expressed as a percentage of control. Effects of CP on MPTP-induced survival-related GDNF signaling. Its factors (GDNF and Ret) were measured by western blotting (d) and ELISA kit (e). Values are presented as means ± S.E.M. ^∗∗∗^*p* < 0.001 compared with the control group and ^#^*p* < 0.05 or ^###^*p* < 0.001 compared with the MPP^+^- or MPTP-treated group.

To investigate whether CP affects mitochondrial dysfunction and mitochondria-mediated apoptosis, we assessed the mitochondrial membrane potential in differentiated PC12 cells and the levels of Bcl-2, Bax, Cyt-c, poly (ADP-ribose) polymerase (PARP), cleaved caspase-9, and cleaved caspase-3 in the mouse SNPC. MPTP caused a significant decrease in Bcl-2 and an increase in Bax, Cyt-c, PARP, cleaved caspase-9, and cleaved caspase-3, while 1, 5, or 25 mg/kg CP increased Bcl-2 and decreased Bax, Cyt-c, PARP, cleaved caspase-9, and cleaved caspase-3 (Figure 7(a)). Green fluorescence (monomeric form, low Δ*ψ*m) and red fluorescence (aggregate form, high Δ*ψ*m) indicate Δ*ψ*m depolarization. MPP^+^-induced toxicity decreased Δ*ψ*m (by 4.64 ± 0.08 ratio), whereas CP pretreatment prevented depolarization of the mitochondrial membrane (by 1.99 ± 0.05 to 1.42 ± 0.07 ratio) (Figures 7(b) and 7(c)).

### 3.7. Effects of CP on MPTP-Induced Expression of Glial Cell Line-Derived Neurotrophic Factor (GDNF) and Ret in the SNPC

To investigate whether CP affects dopaminergic production, we assessed the levels of GDNF and Ret in the mouse SNPC. MPTP significantly decreased the expression of GDNF and Ret, while 5 or 25 mg/kg CP treatment increased the levels of GDNF and Ret (Figure 7(d)). Using an enzyme-linked immunosorbent assay (ELISA) kit, we found that MPTP decreased GDNF in the SNPC (by 1.80 ± 0.54 pg/mL) and ST (by 2.45 ± 1.30 pg/mL), while treatment with 1–25 mg/kg CP reduced the MPTP-induced expression of GDNF in the SNPC (by 3.01 ± 0.63 to 7.51 ± 1.55 pg/mL) and ST (by 3.08 ± 0.56 to 14.30 ± 1.35 pg/mL) (Figure 7(e)).

### 3.8. Effects of CP on MPTP- or LPS-Induced Glial/Microglial Activation and Neuroinflammatory Factor Production

**Figure 8:**
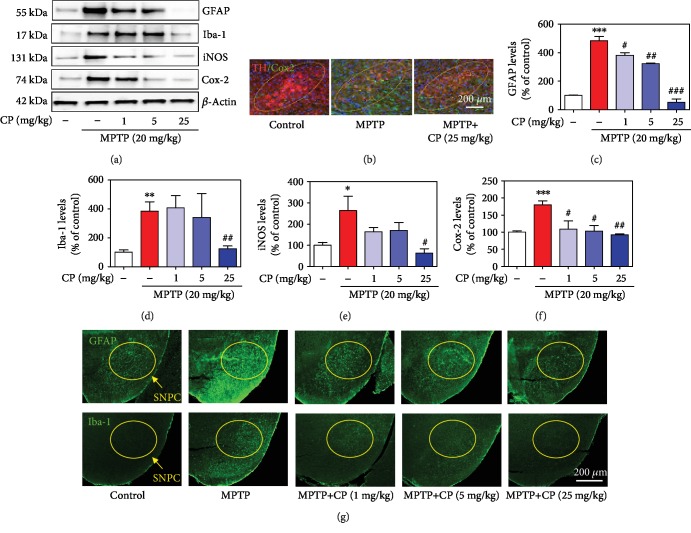
Effects of CP on MPTP-induced neuroinflammatory signaling factors. One day after the last MPTP treatment, glial activation proteins (GFAP and Iba-1) and neuroinflammatory signaling factors (iNOS and Cox-2) were measured by western blotting or IHC (a, b). Levels of neuroinflammatory signaling factors were normalized to *β*-actin (c–f). Moreover, representative photomicrographs of the SNPC were taken (g). Values shown represent means ± S.E.M. ^∗^*p* < 0.05, ^∗∗^*p* < 0.01, and ^∗∗∗^*p* < 0.001 compared with the control group and ^#^*p* < 0.05, ^##^*p* < 0.01, and ^###^*p* < 0.001 compared with the MPTP-treated group.

**Figure 9:**
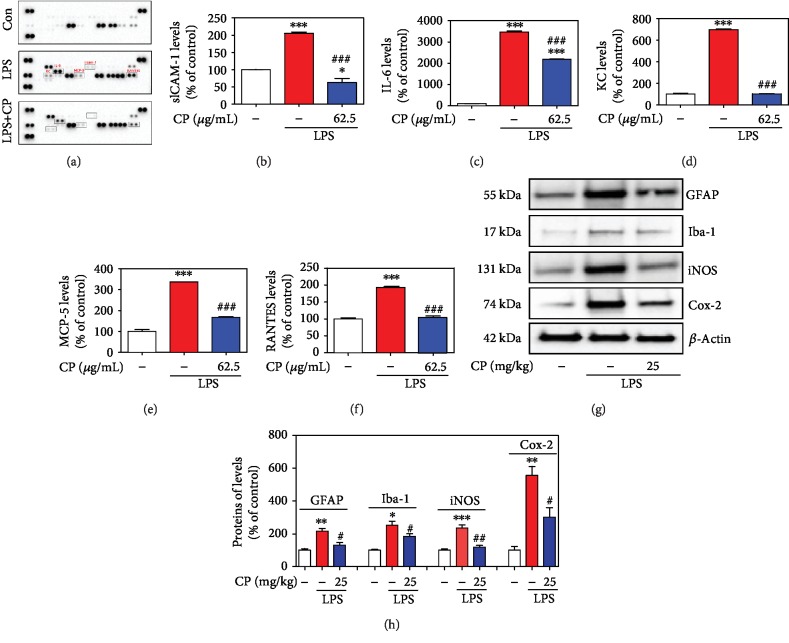
Effects of CP on LPS-induced neuroinflammatory signaling factors. Microglial BV2 cells were treated with CP for 2 h and with LPS for an additional 22 h. After 24 h incubation, the cultures were subjected to a cytokine antibody array assay (h). Densitometric ratios of the arrays showed differences in the cytokine markers (ICAM-1, KC, IL-6, MCP-5, and RANTES) (i–m). The glial activation markers (GFAP and Iba-1) and neuroinflammatory signaling factors (iNOS and Cox-2) were measured using western blotting (n). Bar graphs represent the relative expression of GFAP, Iba-1, iNOS, and Cox-2 (o) for (n). Values shown represent means ± S.E.M. ^∗^*p* < 0.05, ^∗∗^*p* < 0.01, and ^∗∗∗^*p* < 0.001 compared with the control group and ^#^*p* < 0.05, ^##^*p* < 0.01, and ^###^*p* < 0.001 compared with the LPS-treated group.

To investigate whether CP affects the neuroinflammatory response, we assessed glial/microglial activation and the levels of neuroinflammatory factors in microglial BV2 cells and the mouse SNPC. We measured the expression levels of GFAP, Iba-1, iNOS, and Cox-2, which are released following inflammatory stimuli, by western blotting. Western blotting analysis revealed that the levels of GFAP, Iba-1, iNOS, and Cox-2 were significantly increased (by 482.97 ± 28.90%, 384.06 ± 65.22%, 264.07 ± 77.82%, and 179.78 ± 11.50%, respectively) in the SNPC of the MPTP-treated group. In contrast, CP suppressed this decrease in GFAP, Iba-1, iNOS, and Cox-2 (by 381.29 ± 19.15% to 52.02 ± 24.48%, 407.13 ± 85.73% to 124.79 ± 19.71%, 164.17 ± 23.04% to 63.20 ± 24.39%, and 109.09 ± 23.71% to 92.02 ± 2.36%, respectively) (Figures 8(a)–8(f)). Representative photomicrographs were taken of immunofluorescence staining with anti-Cox-2, GFAP, and Iba-1 antibodies in the SNPC (Figures 8(b) and 8(g)). Microglial BV2 cell cytotoxicity was not affected by 1–62.5 *μ*g/mL CP, as assessed by the MTT assay at 24 h after treatment. However, 125–1000 *μ*g/mL CP increased cytotoxicity (data not shown). Thus, all further experiments were performed with CP at 1–62.5 *μ*g/mL. Cytokine array kits revealed that the levels of intercellular adhesion molecule 1 (ICAM-1), interleukin-6 (IL-6), KC, MCP-5, and RANTES were significantly increased (by 205.03 ± 4.24%, 3468.59 ± 51.63%, 687.96 ± 6.04%, 336.64 ± 0.07%, and 192.58 ± 1.65%, respectively) in LPS-treated microglial BV2 cells. In contrast, CP suppressed the increase in ICAM-1, IL-6, KC, monocyte chemotactic protein 5 (MCP-5), and RANTES (by 62.45 ± 12.28%, 2185.72 ± 11.60%, 102.53 ± 2.19%, 168.26 ± 3.22%, and 104.78 ± 0.46%, respectively) (Figures 9(a)–9(f)). The levels of iNOS and Cox-2 were also significantly increased (by 235.75 ± 18.69% and 555.89 ± 55.49%, respectively) in the SNPC of the LPS-treated group. Moreover, CP treatment suppressed this increase in iNOS and Cox-2 (by 118.92 ± 10.06% and 301.61 ± 58.46%) (Figures 9(g) and 9(h)).

## 4. Discussion

Many intrinsic signals and extrinsic transcription factors have been identified to play critical roles in dopaminergic neuronal development in the midbrain in PD. This process depends on two major signaling pathways: (i) Sonic hedgehog/FoxA2 and (ii) wingless-type MMTV integration site family, member 1/Lmx1a, and their downstream signaling molecules [37, 38]. These two signaling pathways merge to control the expression levels of Nurr1, suggesting Nurr1 as a key regulator of dopaminergic neurons [39, 40]. Indeed, dopaminergic neurons fail to develop in mice lacking the Nurr1 receptor [12]. We used the VOSviewer software to visualize network similarities [41, 42]. We found a cluster of keywords across publications related to Nurr1, Alzheimer's disease pathology, PD, dopaminergic neurons, GDNF, alpha-synuclein, canonical ligand-binding pocket, and cilostazol (Supplementary Fig. [Supplementary-material supplementary-material-1]). These results led us to think that Nurr1 may be a promising target in the treatment of PD.

According to the WHO, as much as 80% of the world's population relies primarily on animal- and plant-based medicines [43–45]. Animal-assisted therapy is known as zootherapy (ZT) [45]. The phenomenon of ZT is marked both by a broad geographical distribution and very deep historical origins [45]. Despite its importance, studies on ZT have been neglected, when compared to those on plant-based therapies. However, in modern societies, ZT constitutes an important alternative to other therapies practiced worldwide [45]. Wild and domestic animals and their by-products (e.g., hooves, skins, bones, feathers, and tusks) provide important ingredients to curative, protective, and preventive medicine [46–48]. Recently, there has been increased interest in animal-based medicines, and several animals have been tested by pharmaceutical companies as potential sources of modern drugs [49]. Based on the previous study on Nurr1, we screened a Korean traditional medicine library composed of clinically used drugs (Chung-bu category in *Dongui Bogam*) and identified one hit drug. A previous report showed that Nurr1 promotes dopaminergic neuronal development by inducing the expression of neurotrophic factors, such as TH, DDC, DAT, and VMAT2 [4, 27]. Dopamine is transported into synaptic vesicles by VMAT2, a major factor in maintaining dopamine homeostasis in dopaminergic presynaptic terminals [50, 51]. It has also been demonstrated that Nurr1 gene expression in peripheral blood lymphocytes of patients with PD is decreased compared to that of healthy people [10]. In the present study, CP increased the levels of Nurr1 and its regulating proteins, TH, DDC, DAT, and VMAT2, in differentiated PC12 cells and the mouse SNPC. Moreover, Nurr1 knockdown using siRNA blocked a CP-mediated increase in TH, DAT, and VMAT2 protein expression, suggesting that Nurr1 activation is crucial for CP-induced TH, DAT, and VMAT2 upregulation. Recently, studies have shown that two regions in Nurr1, the N-terminal and C-terminal regions, are important for its transcriptional activation [52]. The Nurr1 N-terminus is important for regulating transcription in a mitogen-activated protein kinase- (MAPK-) dependent manner [52]. It has been reported that Nurr1 can be phosphorylated by ERK1/2 and translocate to the nucleus, where it upregulates TH expression [52–54]. In the present study, an ERK inhibitor inhibited a CP-induced increase in Nurr1, TH, DDC, DAT, and VMAT2 in differentiated PC12 cells. We also found that CP can regulate striatal dopamine levels in the mouse ST. MAO-B is mainly involved in dopamine metabolism [55]. To confirm that the disease-modifying actions of CP could not be ascribed to the prevention of conversion of MPTP to MPP+, we also measured the activity of MAO-B [25, 55]. However, CP had no effect on MAO-B activity. These results suggest that CP increases neurotrophic factors by upregulating Nurr1 expression via ERK phosphorylation. It has been recently suggested that Nurr1 inducers exert neuroprotective effects in experimental models of PD; thus, possible future therapeutic strategies for PD may include inducing Nurr1 signaling by CP.

The above-described findings prompted us to test whether CP can ameliorate motor behavior deficits in a mouse model of MPTP-induced PD. This animal model is used widely because dopaminergic neuronal loss in the SNPC and ST is associated with the onset of motor symptoms, and there is a direct relationship between the extent of dopamine loss and motor dysfunction [28, 56]. The pole test assesses animal agility and includes measures of muscle rigidity and bradykinesia [57]. The rotarod test can be used to assess motor coordination and postural balance [57]. In the pole test, CP-treated mice showed a significant improvement in T-turn and T-LA, with measurements similar to those in the control group. In addition, in the rotarod test, CP prolonged the duration spent by the mice on the rotarod. Similar to our study, Hsieh et al. reported that CP increases hypomotility induced by a TH inhibitor or 5-hydroxytryptophan (a precursor of serotonin) [21]. Moreover, our results showed that CP significantly increased MPTP-induced dopamine levels. CP can stabilize central catecholaminergic and serotonergic activity and is expected to be effective in various disorders, such as convulsion and insomnia [21]. Therefore, we think there is a need to focus on further studies on catecholamine and behavioral changes. Furthermore, we confirmed these effects by TH-specific IHC, which demonstrated that CP protected both dopaminergic neurons in the SNPC and their fibers in the ST, compared with mice treated with MPTP only. In the present study, the number of TH/Nurr1 double-positive neurons and Nurr1 and its regulating proteins in the SNPC declined in the MPTP group compared with the control group, while colocalization and expression levels were increased in the CP-treated groups. GDNF and its canonical receptor Ret can signal together or independently to fulfill many important functions in the midbrain's dopaminergic system [58, 59]. Nurr1 and Ret, its downstream target, were found to be transcriptionally downregulated by *α*-synuclein accumulation [60]. Reduced Ret protein levels might prevent GDNF-induced survival response in midbrain dopaminergic neurons [61]. Thus, this rationale linked GDNF/Ret signaling to Nurr1, another protein found to be mutated in a rare familial form of PD (autosomal recessive loss-of-function mutation). In this study, CP significantly protected MPTP-induced GDNF/Ret signaling in mice. Taken together, the present study showed that CP significantly improved MPTP-induced PD-like movement problems and protected against dopaminergic neuronal damage and neurotrophic response activity in mouse dopaminergic neurons.

Finally, because Nurr1 has proapoptotic as well as antiapoptotic effects, we also analyzed the effects of CP on mitochondrial dysfunction and mitochondria-mediated apoptosis. The Bcl-2 protein family contains key apoptosis-regulating proteins that can promote cell survival or induce cell death [62]. Bcl-2 appears to directly or indirectly preserve the integrity of the outer mitochondrial membrane, thus preventing Cyt-c release and mitochondria-mediated cell death initiation [62, 63]. On the other hand, the proapoptotic protein Bax promotes Cyt-c release from the mitochondria, with subsequent cleaved caspase-9/caspase-3 and PARP [63]. Recently, Nurr1 has been shown to downregulate the expression of the proapoptotic protein Bax, which is directly transactivated by the tumor suppressor p53 [64]. Microarray analysis revealed that overexpression of Nurr1 downregulates cleaved caspase-3 and other apoptotic factors in neural stem cells [65]. In this study, MPTP induced a slight decrease in Bcl-2 expression and an increase in Bax expression; CP protected against these changes. Moreover, MPTP-induced toxicity increased Cyt-c, whereas CP inhibited Cyt-c release. The induction of Cyt-c-mediated cleaved caspase-9/caspase-3 and PARP levels by proapoptotic agents, including MPTP, appears to be essential for apoptosis, and treatment with CP prevented the MPTP-induced increase in cleaved caspase-9/caspase-3 and PARP levels. Because Nurr1-induced antiapoptotic effects are associated with pro- as well as anti-inflammatory responses, we also checked the effects of CP on proinflammatory cytokines and signaling molecules. Moreover, Xu et al. demonstrated that CP can decrease inflammatory-related proteins such as IL-6, iNOS, and Cox-2 in LPS-induced Raw 264.7 macrophage cells [20]. Moreover, according to Chang et al., CP inhibits inflammation-related proteins such as IL-6 and NF-*κ*B via modulating reactive oxygen species induced by ultraviolet B irradiation on keratinocyte HaCaT cells [23]. Therefore, it was possible to have an anti-inflammatory effect. In our study, CP repressed neuroinflammatory signaling molecules, such as iNOS and Cox-2, and glial/microglial activation in MPTP- or LPS-treated mice. Moreover, when microglial cells were treated with inflammation-inducing LPS for 24 h, the expression of proinflammatory genes (cytokine array: G-CSF, GM-CSF, sICAM-1, IL-1ra, IL-2, IL-3, IL-5, IL-6, IL-7, IL-12 p70, IL-16, IL-17, IL-23, IP-10, I-TAC, KC, M-CSF, MCP-5, MIP-2, RANTES, SDF-1, TARC, and TNF-*α*) was increased more than 2-fold. Remarkably, CP reduced the expression of ICAM-1, KC, IL-6, MCP-5, and RANTES (>30%). However, further studies using Nurr1 transgenic mice will be required to confirm our findings. Next, we analyzed protein-protein interactions using the STRING database and potential molecular mechanisms using the KEGG database (Supplementary Fig. [Supplementary-material supplementary-material-1]). We found functional relationships among the proteins primarily related to inflammatory and neuronal death mechanisms, including small-cell lung cancer, tuberculosis, AGE-RAGE signaling pathway in diabetic complications, apoptosis, amyotrophic lateral sclerosis, hepatitis B, influenza A, platinum drug resistance, pathways in cancer, colorectal cancer, Kaposi's sarcoma-associated herpesvirus infection, toxoplasmosis, TNF signaling pathway, PD, legionellosis, and p53 signaling pathway. Taken together, our data showed that CP enhanced the dual role of Nurr1: CP (i) increased the expression of Nurr1 and its regulating proteins in dopaminergic neurons and (ii) inhibited mitochondria-mediated apoptosis and proinflammatory cytokine gene expression.

This study has some limitations. First, we did not investigate the effects of CP on MPTP-induced PD in mice lacking Nurr1. Studies using Nurr1-lacking mice are being carried out; however, more research is still needed because these studies are in their screening stages. Therefore, we confirmed it using siRNA as a pilot study, and we believe that more detailed research is needed through knockout animal models. Second, doses of CP in human were 3-6 g daily [15, 16]. According to human equivalent dose calculation, for body weight of 60 kg, the corresponding human dose of the extract of CP was 121.8 mg/day. Among our unpublished studies, we evaluated the safety of the CP extract using a standard toxicological study design to assess the potential oral dose toxicity. During the study period of 2 weeks, C57BL/6 mice were orally administered once daily with doses of 50, 150, or 450 mg/kg/day of the CP extract after which several study parameters of mortality, clinical signs, changes in body weight, gross findings, organ weight, histopathological examinations, and hematology were assessed. We demonstrated that the CP extract did not have any adverse effects in mice up to a dose of 450 mg/kg/day for a 2-week administration period (data not shown). The human equivalent dose calculated as 121.8 mg/day is 18-fold lower than the 450 mg/kg/day (2189.1 mg/day in human) of the CP extract used in our unpublished study. Therefore, this study provides an important reference for the safety of the CP extract for humans. However, more detailed research on clinicians will not be available until further studies are conducted. Lastly, we did not identify the active compounds of CP that are responsible for its anti-PD effects. In this study, we mainly analyzed and separate catecholamine compounds, focusing on the various neurohormonal changes in insect metamorphosis (Supplementary Fig. [Supplementary-material supplementary-material-1]) [66–68]. This is because we expected a large amount of neurohormones to be released and left in the skin when the insect was exterminated. However, since this study was only a pilot, screening study, we think that more research is needed to reveal more accurate mechanisms. Future studies are needed to analyze the specific contribution of the various active compounds of CP.

**Figure 10:**
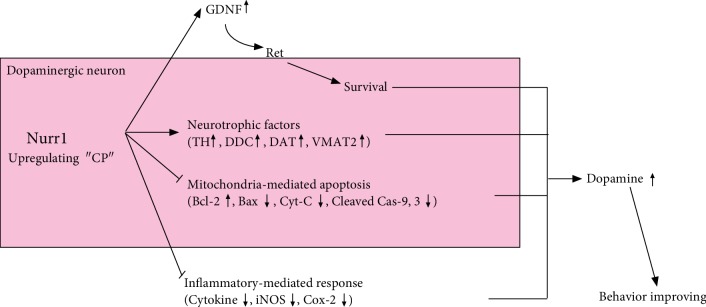
Schematic of the mechanism proposed for the effects of CP on Nurr1 activation and Parkinson's disease pathogenesis.

In conclusion, we demonstrated that CP increased the expression of Nurr1 and its regulating proteins (TH, DDC, DAT, and VMAT2) in vitro and in vivo (Figure 10). Further, CP protected dopaminergic neurons against MPTP-induced neurotoxicity via regulating mitochondria-mediated apoptotic molecules, such as Bcl-2, Bax, Cyt-c, cleaved caspase-9, caspase-3, and PARP; neuroinflammatory signaling molecules, such as cytokines, iNOS, and Cox-2; and glial/microglial activation. Our findings suggest that CP might contribute to neuroprotective signaling by regulating neurotrophic factors via Nurr1, mitochondria-mediated apoptosis, and neuroinflammation.

## Data Availability

The data used to support the findings of this study are available from the corresponding author upon request.

## Supplementary Materials

Supplementary Materials Supplementary Fig. 1: effects of PC on MPTP-induced Nurr1 levels. Seven days after the last MPTP treatment, Nurr1 levels were measured by immunofluorescence in dopaminergic neurons of SNPC. Supplementary Fig. 2: keyword cluster network (A) and density visualization (B) of articles published in 2002–2019. Supplementary Fig. 3: a network of clusters and functional relationships from proteins, generated using the STRING software. Supplementary Fig. 4: LC-MS analysis of PC. UPLC chromatogram monitored at 280 nm. Supplementary Table 1: list of antibodies. Supplementary Table 2: list of primer sequences for real-time RT-PCR.
