# Supplementary material for: Cicadidae Periostracum, the Cast-Off Skin of Cicada, Protects Dopaminergic Neurons in a Model of Parkinson's Disease
Source: Oxid Med Cell Longev. 2019 Oct 24;2019:5797512. doi: 10.1155/2019/5797512 (PMC6854990; doi:10.1155/2019/5797512)
Supplement: Supplementary Materials — Supplementary Fig. 1: effects of PC on MPTP-induced Nurr1 levels. Seven days after the last MPTP treatment, Nurr1 levels were measured by immunofluorescence in dopaminergic neurons of SNPC. Supplementary Fig. 2: keyword cluster network (A) and density visualization (B) of articles published in 2002–2019. Supplementary Fig. 3: a network of clusters and functional relationships from proteins, generated using the STRING software. Supplementary Fig. 4: LC-MS analysis of PC. UPLC chromatogram monitored at 280 nm. Supplementary Table 1: list of antibodies. Supplementary Table 2: list of primer sequences for real-time RT-PCR. [file 5797512.f1.pdf]

Supplementary Data

Cicadidae Periostracum, the cast-off skin of cicada, protects dopaminergic neurons in a model of Parkinson's disease

Running title: Cicadidae Periostracum protects dopaminergic neurons

Hye-Sun Lim[1*], Joong-Sun Kim[1], Byeong Cheol Moon[1], Goya Choi[1], Seung Mok Ryu[1], Jun Lee[1], Mary Jasmin Ang[2], Mijin Jeon[2], Changjong Moon[2], and Gunhyuk Park[1¶]

[1]*Herbal Medicine Resources Research Center, Korea Institute of Oriental Medicine, 111 Geonjae-ro, Naju-si, Jeollanam-do, 58245, Republic of Korea*

[2]*College of Veterinary Medicine and BK21 Plus Project Team, Chonnam National University, Gwangju 61186, Republic of Korea*

[¶]**Corresponding author:** Gunhyuk Park, Ph.D.

Senior Researcher, Herbal Medicine Resources Research Cent er, Korea Institute of Oriental Medicine, 111 Geonjae-ro, Naju-si, Jeollanam-do, 58245, Republic of Korea

Tel.: +82-61-338-7112

E-mail address: *gpark@kiom.re.kr* and *parkgunhyuk@gmail.com*

Supplementary Materials

Materials and Methods

Data collection and scientometric analysis

The Science Citation Index Expanded citation index databases in the Web of Science core collection, a common data source for scientific and bibliometric research, were retrieved as the source database for this study. The search expressions included the application of "PD" to the "Nurr1" field, which retrieves the title, abstract, and keywords of publications in Web of Science to find the required literature. The time span was "2002 to 2019", and the publication types selected were "Article" and "Review" because they represent most of the literature with complete research results. After the search, a total of 335 eligible documents were collected, and the search results were saved as "Other File Formats" with "Full Record and Cited References" on 20 March 2019.

Data analyses

We used the Search Tool for the Retrieval of Interacting Gene (STRING) software to construct a network model showing protein interactions based on known and predicted protein–protein interactions. In addition, protein ontology and functional relationships were obtained from the Kyoto Encyclopedia of Genes and Genomes (KEGG) database. The main research method of this paper was based on Scientometrics, and analysis software included the Visualization of Similarities viewer (VOSviewer; developed by van Eck and Waltman of Leiden University in The Netherlands) (version 1.6.9; www.vosviewer.com) and text files. VOSviewer can analyze complex networks using clustering analysis according to the connection strength between projects. It has strong advantages such as mapping knowledge domain display, especially clustering; in analyzing large-scale data sets; and constructing complex networks.

Liquid chromatography-mass spectrometry (LC-MS) analysis

LC-MS was performed using an Acquity Ultra high-performance liquid chromatography system (Waters, Milford, MA, USA) and LCQ Fleet Ion Trap mass spectrometer (Thermo Scientific, Madison, WI, USA). Chromatographic separation was performed using an Acquity UPLC BEH C18 column (inner diameter, 2.1 × 100 mm, 1.7 µm). The mobile phase consisted of water containing 0.5% formic acid and acetonitrile using a gradient of 5−50 % for 0−15 min with a flow rate of 300 µL/min. The MS spectrometer parameters were as follows: nebulizing and sheath nitrogen gas pressure, 60 and 20 psi; capillary temperature, 200 °C; and capillary voltage, −26 V.

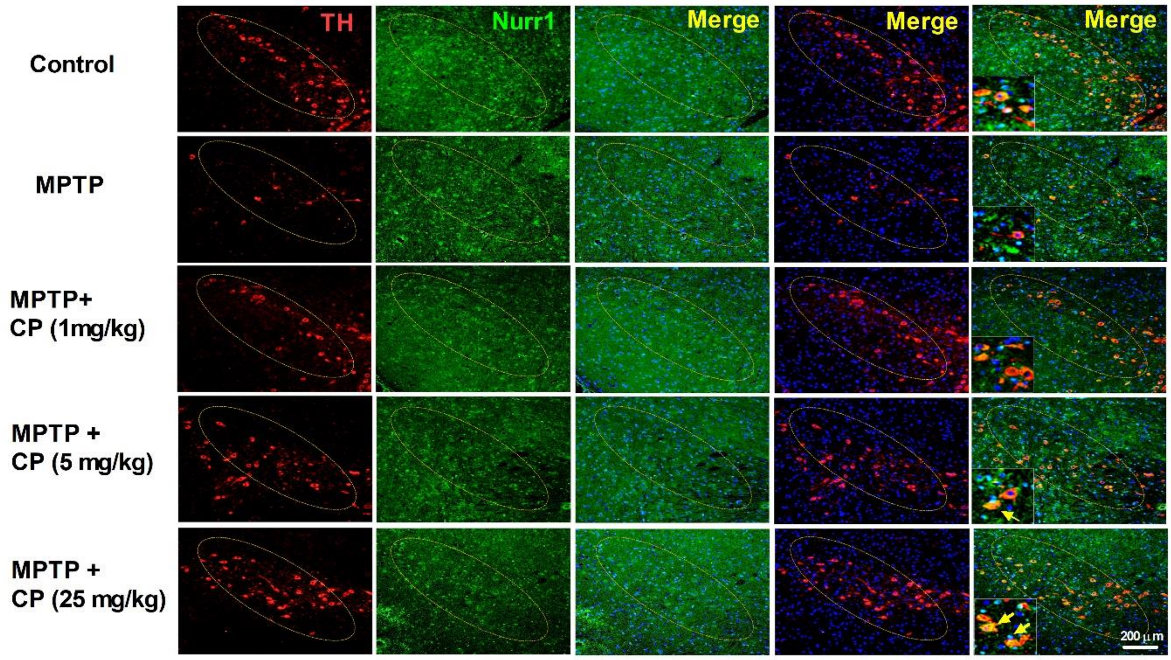

Supplementary Fig. 1 Effects of CP on MPTP-induced Nurr1 levels. Seven days after the last MPTP treatment, Nurr1 levels were measured by immunofluorescence in dopaminergic neurons of SNpc.

(A)

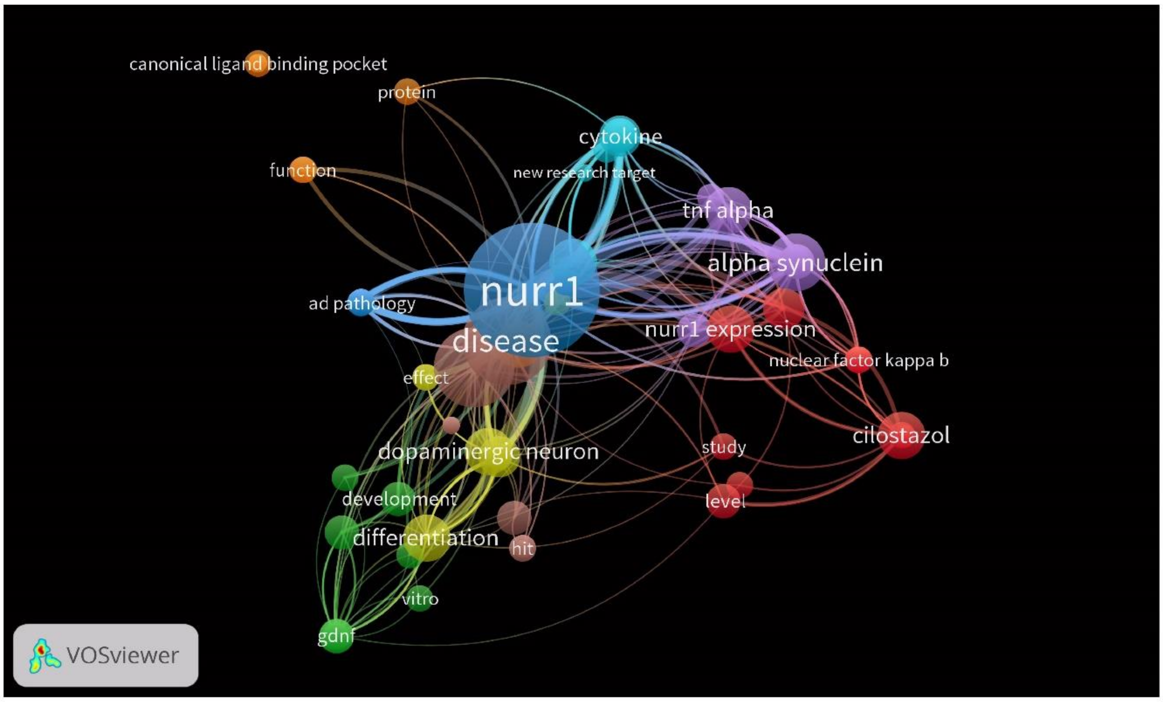

(B)

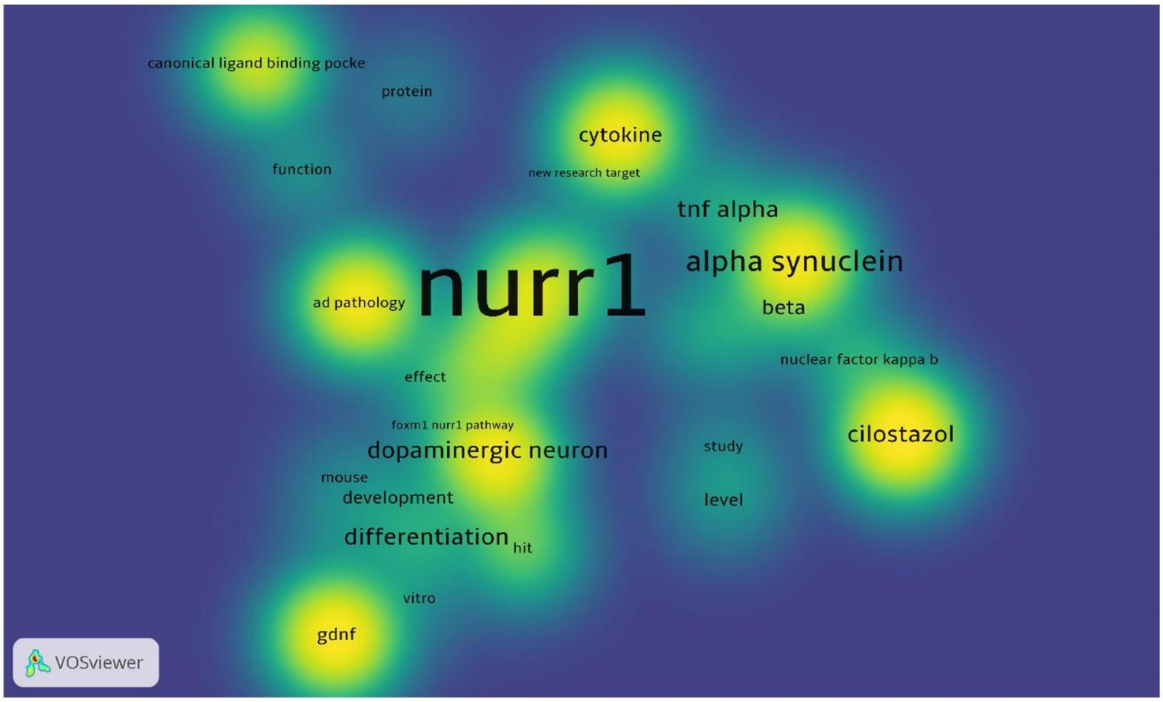

Supplementary Fig 2. Keywords cluster network (A) and density visualization (B) of articles published in 2002–2019.

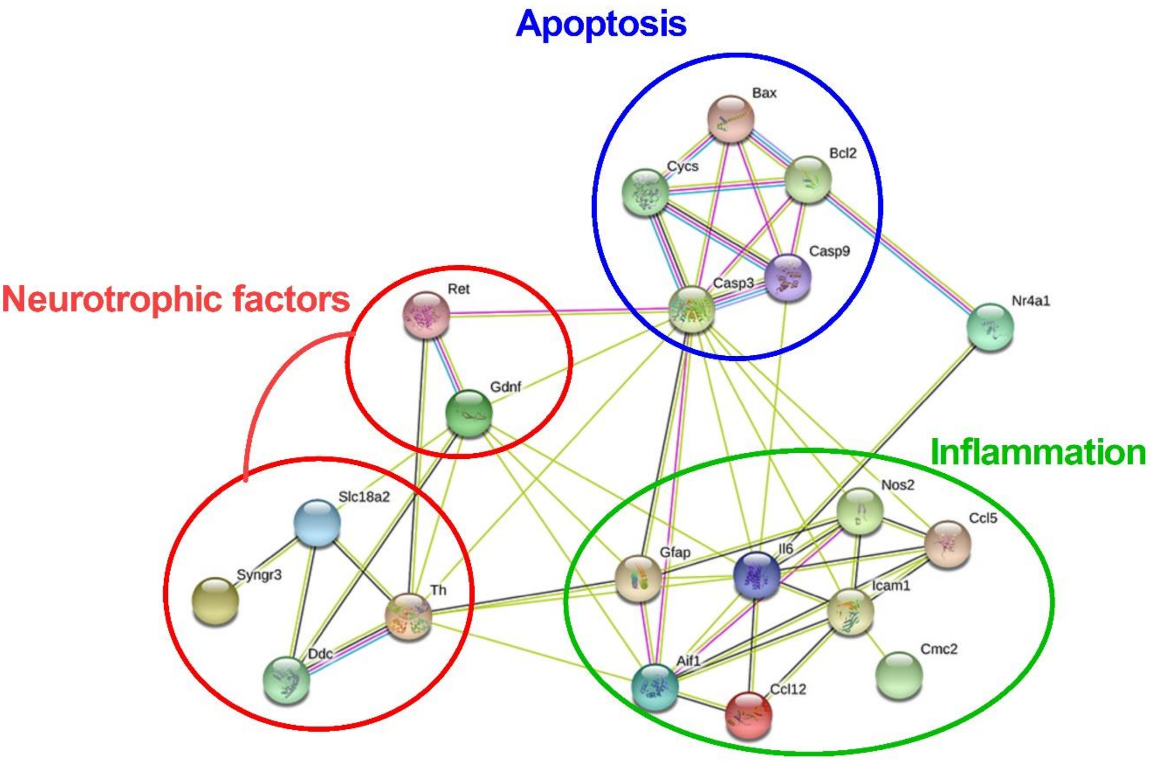

Supplementary Fig 3. A network of clusters and functional relationships from proteins, generated using STRING software

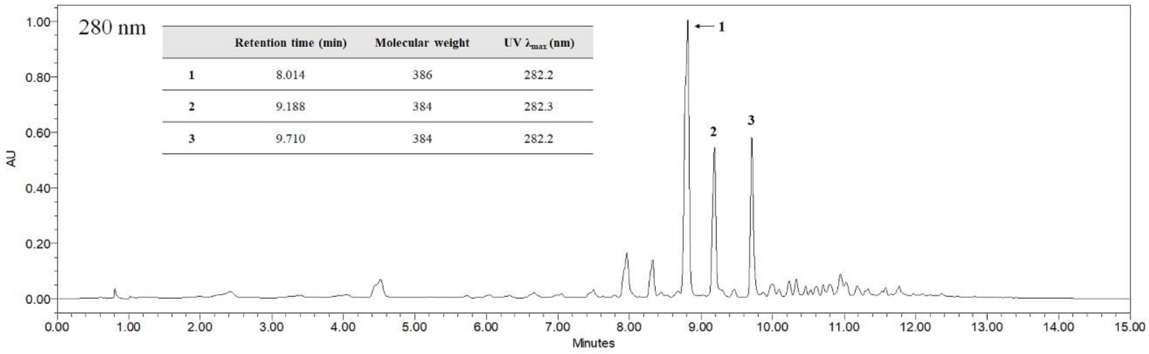

Supplementary Fig 4. LC-MS analysis of CP. UPLC chromatogram monitored at 280 nm.

Supplementary Table 1. List of antibodies.

Primary antibody	Host	Company	Catalog#
Tyrosine hydroxylase (TH)	Mouse	Millipore	MAB318
	Rabbit	Millipore	AB152
Nuclear receptor-related 1 (Nurr1)	Rabbit	Origene	TA309774
DOPA decarboxylase (DDC)	Rabbit	Abcam	AB3905
Dopamine transporter (DAT)	Rat	Santa Cruz Biotechnology	SC-32258
Vesicular monoamine transporter (VMAT2)	Rabbit	Santa Cruz Biotechnology	SC-15314
β-actin (β-actin)	Mouse	Santa Cruz Biotechnology	SC-47778
B-cell lymphoma 2 (Bcl-2)	Rabbit	Santa Cruz Biotechnology	SC-783
Bcl-2-associated X (Bax)	Rabbit	Abcam	AB7977
Cytochrome-C (Cyt-C)	Mouse	Santa Cruz Biotechnology	SC-13156
Cleaved caspase-9	Mouse	Cell Signaling	#9509
Cleaved caspase-3	Rabbit	Cell Signaling	#9661
Poly [ADP-ribose] polymerase 1 (PARP1)	Mouse	Santa Cruz Biotechnology	SC-8007
Glial cell-derived neurotrophic factor (GDNF)	Mouse	Santa Cruz Biotechnology	SC-13147
	Rabbit	Alomone Labs	ANT-014
	Rabbit	Abcam	AB18956
Ret	Mouse	R&D Systems	AF482
Glial fibrillary acidic protein (GFAP)	Goat	Santa Cruz Biotechnology	SC-6170
Allograft inflammatory factor 1 (Iba-1)	Rabbit	Wako	019-19741
	Rabbit	Wako	016-20001
Nitric oxide synthase (iNOS)	Rabbit	Thermo Fisher Scientific	PA1-036
Cyclooxygenase-2 (Cox-2)	Rabbit	Santa Cruz Biotechnology	SC-1747
**Secondary antibody**		**Company**	**Catalog#**
Biotinylated goat anti-mouse IgG		Vector Laboratories	BA-9200
Biotinylated goat anti-rabbit IgG		Vector Laboratories	BA-1000
Biotinylated goat anti-rat IgG		Vector Laboratories	BA-9400
Biotinylated horse anti-goat IgG		Vector Laboratories	BA-9500
Alexa Fluor® 594		Thermo Fisher Scientific	A11032
Alexa Fluor® 488		Thermo Fisher Scientific	A11008
Goat anti-rabbit IgG-HRP		Santa Cruz Biotechnology	SC-2030
Goat anti-mouse IgG-HRP		Santa Cruz Biotechnology	SC-2061
Bovine anti-goat IgG-HRP		Santa Cruz Biotechnology	SC-2378

Supplementary Table 2. List of primer sequences for real-time RT-PCR.

Gene	Sequences of primers (5' to 3')		Annealing (°C)
	Forward	Reverse	
**Nurr1**	GCAGACTTTAGGTGCATGTTGG	CCCGTCAGATCTCCTTGTCC	59
**TH**	CAGAAGAGCCGTCTCAGAGC	CCTCGAATACCACAGCCTCC	59
**DAT**	TGTATGTGGTCGTGGTCAGC	GGCCAGTTTCTCTCGGAAGG	59
**VMAT2**	TGTGACCAACACGACTGTCC	CAAGAGGAGCCGATTCCCTG	59
**GAPDH**	CCCTTAAGAGGGATGCTGCC	ACTGTGCCGTTGAATTTGCC	59
